# Ulk1, Not Ulk2, Is Required for Exercise Training-Induced Improvement of Insulin Response in Skeletal Muscle

**DOI:** 10.3389/fphys.2021.732308

**Published:** 2021-09-30

**Authors:** Joshua C. Drake, Rebecca J. Wilson, Di Cui, Yuntian Guan, Mondira Kundu, Mei Zhang, Zhen Yan

**Affiliations:** ^1^Department of Medicine, University of Virginia School of Medicine, Charlottesville, VA, United States; ^2^Center for Skeletal Muscle Research, The Robert M. Berne Cardiovascular Research Center, University of Virginia School of Medicine, Charlottesville, VA, United States; ^3^Department of Pharmacology, University of Virginia School of Medicine, Charlottesville, VA, United States; ^4^Department of Biochemistry, University of Virginia School of Medicine, Charlottesville, VA, United States; ^5^Department of Pathology, St. Jude Children’s Research Hospital, Memphis, TN, United States; ^6^Department of Molecular Physiology and Biological Physics, University of Virginia School of Medicine, Charlottesville, VA, United States

**Keywords:** autophagy, exercise, skeletal muscle, insulin signaling, Unc51 like autophagy activating kinase

## Abstract

Unc51 like autophagy activating kinase 1 (Ulk1), the primary autophagy regulator, has been linked to metabolic adaptation in skeletal muscle to exercise training. Here we compared the roles of Ulk1 and homologous Ulk2 in skeletal muscle insulin action following exercise training to gain more mechanistic insights. Inducible, skeletal muscle-specific *Ulk1* knock-out (Ulk1-iMKO) mice and global *Ulk2* knock-out (Ulk2^–/–^) mice were subjected to voluntary wheel running for 6 weeks followed by assessment of exercise capacity, glucose tolerance, and insulin signaling in skeletal muscle after a bolus injection of insulin. Both Ulk1-iMKO and Ulk2^–/–^ mice had improved endurance exercise capacity post-exercise. Ulk1-iMKO did not improve glucose clearance during glucose tolerance test, while Ulk2^–/–^ had only marginal improvement. However, exercise training-induced improvement of insulin action in skeletal muscle, indicated by Akt-S473 phosphorylation, was only impaired in Ulk1-iMKO. These data suggest that Ulk1, but not Ulk2, is required for exercise training-induced improvement of insulin action in skeletal muscle, implicating crosstalk between catabolic and anabolic signaling as integral to metabolic adaptation to energetic stress.

## Introduction

Regular exercise is the most robust and reproducible means for promotion of whole-body health (Drake et al., 2016). Skeletal muscle is particularly integral to the metabolic benefits of regular exercise as it is the primary tissue for the use and storage of glucose. Thus, regular exercise prevents development of metabolic diseases, such as Type II Diabetes, but can also reverse the pathology in those already affected (Cartee et al., 2016). However, the mechanisms underlying the adaptations in skeletal muscle that promote metabolic health remain incompletely understood.

Improved mitochondrial quality (i.e., overall functionality) within skeletal muscle is one result of exercise training that promotes metabolic heath (Laker et al., 2014; Drake et al., 2016). Mitochondrial quality is maintained through synergistic processes that produce and incorporate new mitochondrial proteins and lipids (biogenesis), reorganize the reticulum (dynamics), as well as remove and recycle damaged/dysfunctional regions (mitophagy) (Drake et al., 2016). While all aspects of mitochondrial quality control are sensitive to nutrient status and exercise-induced energetic stress (Drake et al., 2016), the degree to which impairment in mitochondrial quality control contributes to the etiology of metabolic pathologies (e.g., insulin resistance, type II diabetes, etc.) is debated.

Previously, we demonstrated that endurance exercise promotes the removal of damaged/dysfunctional mitochondria through mitophagy (Laker et al., 2014, 2017; Drake et al., 2016). Our data indicated that exercise-induced mitophagy in skeletal muscle is regulated by the energetic sensor 5′ AMP-activated protein kinase (AMPK) (Laker et al., 2017). The energetic stress of acute exercise activates AMPK, which promotes mitophagy by phosphorylating Unc51 like autophagy activating kinase 1 (Ulk1) at Ser555 (Egan et al., 2011; Laker et al., 2017), initiating the mitophagy cascade. Ablation of *Ulk1* in skeletal muscle in tamoxifen-inducible knockout mice ameliorated improvements in glucose clearance observed in wild type mice post-exercise training (Laker et al., 2017), indicative of improved insulin action and a common metabolic benefit of exercise training. However, it is unclear how skeletal muscle Ulk1 influences glucose tolerance as a result of exercise training. As mitophagy, and the more generalizable autophagy, is impaired in numerous pathologies (Choi et al., 2013; Sun et al., 2015; Drake and Yan, 2017), including metabolic disorders (Singh et al., 2009; Yang et al., 2010; Zabielski et al., 2016), insight into the requirement of Ulk1 for metabolic adaptation to exercise could lead to novel therapeutic targets for clinical intervention. Therefore, the aim of the current study was to investigate the insulin-dependent signaling response in skeletal muscle following exercise training in inducible, skeletal muscle-specific *Ulk1* knockout mice (*Ulk1-iMKO*) and homologous *Ulk2* knockout mice (*Ulk2*^–/–^).

## Research Design and Methods

### Animals

All experimental procedures were approved by the University of Virginia, Institutional Animal Care and Use Committee. *Ulk1-iMKO* (*Ulk^*f/f*^; HAS-MerCreMer*) and the wild type littermate mice (*Ulk1^*f*/f^*) were obtained by crossbreeding between loxP-floxed *Ulk1* mice (*Ulk1^*f*/f^*) and *HAS-MerCreMer* mice (generous gift of Dr. Karyn Esser) (Laker et al., 2017). Only male mice were used for the current study. At 9–10 weeks of age, *Ulk1-iMKO* mice and the WT littermates were injected intraperitoneally with tamoxifen (1 mg/25 mg body weight) once daily for 5 days to delete the *Ulk1* gene in skeletal muscle. One Ulk1-iMKO mouse was excluded due to incomplete knock-out of Ulk1, as evidence by western blot. Generation of *Ulk2*^–/–^ mice has been described elsewhere (Cheong et al., 2011). All mice were housed in temperature-controlled (21°C) quarters with 12:12 h light-dark cycle and *ad libitum* access to water and chow (Purina). Importantly, knock-out of either Ulk1 or Ulk2 has been demonstrated to not influence the non-targeted Ulk species (Cheong et al., 2011; Wang et al., 2018).

### Exercise Training

Mice were randomly assigned to either exercise training group, where they were housed individually in cages with free access to running wheels, or to sedentary group where they were housed in cages not equipped with running wheels at 10–11 weeks of age. All mice were provided food and water *ad libitum*. Daily running distance was recorded via computer monitoring.

### Glucose Tolerance Test

After 4 weeks of voluntary wheel running, running wheels were locked for 24 h for the exercise group, and all mice were fasted for the final 6 h. Blood glucose was measured from the tail vein prior to i.p. injection of sterilized D-glucose (2 mg/g) and at 30-, 60-, and 120-min post. Following the test, mice in the exercise group were allowed to resume their running activity for another week.

### Exercise Capacity Test

In the last week of the experiment, mice were acclimatized to treadmill running. Each mouse ran on a treadmill in the morning for 10 min at a speed of 13 m/min at 0% grade for 3 consecutive days. Running wheels in the exercise group were then locked before the exercise capacity test on the fourth day. For the exercise capacity test, mice ran at 13 m/min for 30 min at a 5% grade followed by an increase in running speed of 3 m/min every 30 min until they show perceived exhaustion as reported previously (Laker et al., 2017). Blood lactate was measured prior and immediately after exercise cessation.

### Insulin Injection and Tissue Harvesting

At the end of 6 weeks of training (16–18 weeks of age), running wheels were locked overnight for mice in the exercise group, and all mice were fasted (12 h). The next morning (8 am), all mice were weighed and anesthetized under oxygenated isoflurane. Plantaris skeletal muscle of one hindlimb was harvested and homogenized in 2× Laem mli Sample Buffer containing protease and phosphatase inhibitors (Sigma). Immediately following removal of the plantaris muscle (predominately comprised of type II fibers), the limb was clamped to prevent blood loss. Next, mice were injected intraperitoneally with insulin (5 U/kg), and the contralateral plantaris was harvested 10 min later and processed immediately in the same way (Shao et al., 2000, 2002; Dungan et al., 2014, 2019; Cui et al., 2020). Muscle lysates were then boiled at 97°C for 5 min before being frozen at −80°C until later analysis.

### Western Blotting

Whole plantaris muscle homogenates were run on a 10% gel and transferred to a nitrocellulose membrane. Equal loading was confirmed post-transfer via Ponceau S Red staining and by probing for Gapdh. Membranes were cut to contain desired molecular ranges, depending on the predicted molecular weight of the proteins of interest, and then probed with specific antibodies. The following primary antibodies were used: S473 Akt (CST #9271; 1:500 dilution), Akt (CST #4691; 1:500 dilution), and Gapdh (CST #2118; 1:1,000 dilution). All antibodies were sourced from rabbit and goat anti-rabbit IR800 (LICOR) secondary antibodies were used. Membranes were scanned by using an Odyssey infrared imaging system (LICOR), and densitometry was performed to calculate pAkt (S473)/Akt ratio as an index of insulin-stimulated Akt phosphorylation. Prior to phospho/total calculations, all targets were analyzed in comparison to a common protein standard (CS) loaded on each gel to account for any difference in transfer efficiency between blots (Laker et al., 2017). The common protein standard (CS) consisted of whole tissue lysate mixture of liver, heart, and skeletal muscle.

### Statistics

**FIGURE 1:**
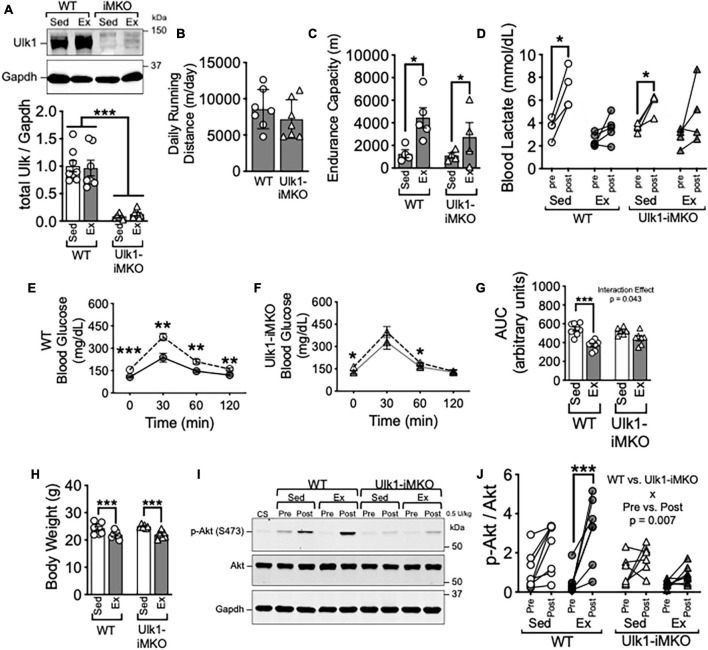
Ulk1 is required for exercise training-induced improvement of insulin sensitivity in skeletal muscle. **(A)** Western blot of Ulk1 protein in skeletal muscle of Ulk1-iMKO and WT mice following 5 days of intraperitoneal injection of tamoxifen; **(B)** average daily running distance in Ulk1-iMKO and WT mice over 6 weeks of voluntary running; **(C)** treadmill running test in exercise and sedentary groups (*n* = 4–5 per group). **(D)** Blood lactate in tail vein blood prior to and immediately after treadmill running test. **(E,F)** Blood glucose prior to and 30-, 60-, and 120-min. following I.P. injection of glucose; **(G)** blood glucose area under the curve during glucose tolerance test; **(H)** body weight at time of tissue harvest in exercise and sedentary groups. **(I)** Western blot of p-Akt (S473) and Akt in whole muscle homogenates of plantaris muscles before and 10 min after intraperitoneal injection of insulin (0.5 U/kg). **(J)** Quantification of western blots in panel **(I)**. WT Sed *n* = 8; WT Ex *n* = 7/ Ulk1-iMKO Sed *n* = 6; Ulk1-iMKO Ex *n* = 7. *, **, and *** denote *p* < 0.05, *p* < 0.01, and *p* < 0.001, respectively. Significant interaction effect from respective two-way ANOVA is stated in the graph.

**FIGURE 2:**
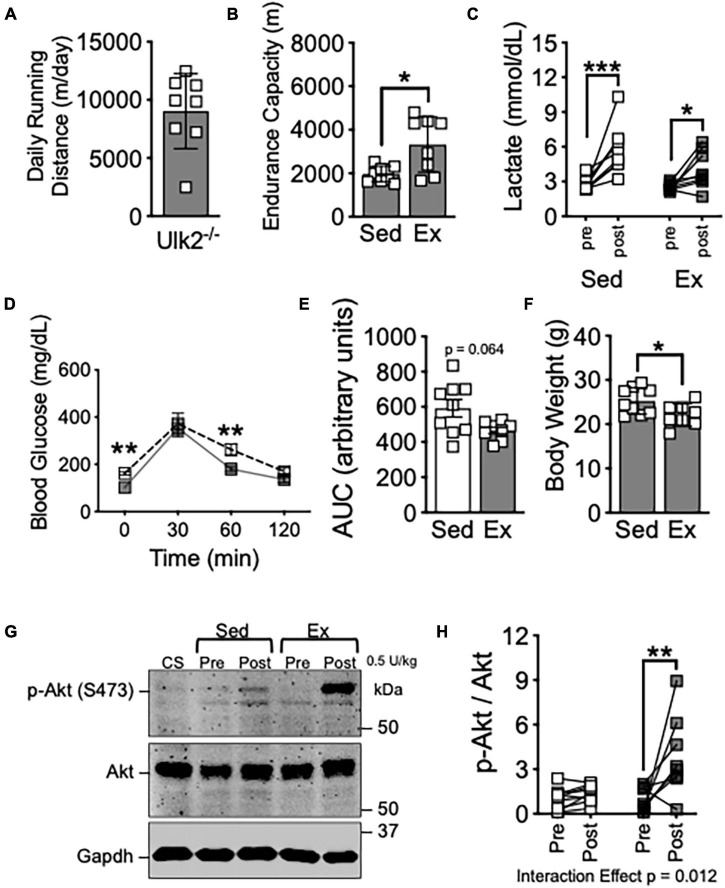
Loss of Ulk2 does not impair exercise training-induced improvement of insulin sensitivity in skeletal muscle. **(A)** Average daily running distance in Ulk2^–/–^ mice; **(B)** treadmill running test in exercise and sedentary groups; **(C)** blood lactate in tail vein blood prior to and immediately after treadmill running test; **(D)** blood glucose prior to and 30-, 60-, and 120-min. following I.P. injection of glucose tolerance test; **(E)** blood glucose area under the curve during glucose tolerance test; **(F)** body weight at harvest following in exercise and sedentary groups; **(G)** western blot of p-Akt (S473) and Akt in whole muscle homogenates of plantaris muscles before and 10 min after intraperitoneal injection of insulin (0.5 U/kg). **(H)** Quantification of western blots in panel **(G)**. *n* = 8–9. *, **, and *** denote *p* < 0.05, *p* < 0.01, and *p* < 0.001, respectively. Significant interaction effect from two-way ANOVA is stated in graph **(H)**.

Data are presented as the mean ± SEM. Student’s *t*-test was used to analyze data with a single variable (Figure 1B, time points in Figures 1E,F and Figure 2B, time points in Figures 2D,E). Lactate data was assessed by Student’s *t*-test as only the pre/post comparison within a given group was considered relevant as a biologic marker of exercise capacity. Data where two variables were considered were analyzed via two-way ANOVA (Figures 1A,C,G, 2H). Significant interaction effects, where found, are indicated on the graph, followed by Tukey *post-hoc* analysis and findings illustrated on the respective graph. Statistical significance was established *a priori* as *p* < 0.05.

## Results

Tamoxifen injection resulted in almost complete loss in detection of Ulk1 by western blot (Figures 1A,B). Average daily running was not different between Ulk1-iMKO and their WT littermates over the entire course of the training period (Figure 1C). Furthermore, endurance exercise capacity post-exercise training increased in a subset of both Ulk1-iMKO and WT mice (Figures 1D,E), as we have shown previously (Laker et al., 2017).

Glucose tolerance was significantly improved following exercise training in WT mice (Figures 1E,G). However, improvement in glucose tolerance post-exercise training was attenuated in Ulk1-iMKO mice (Figures 1F,G), as we have previously shown (Laker et al., 2017), and reiterates the importance of skeletal muscle Ulk1 in mediating exercise training-induced metabolic adaptation. Despite the disparity in glucose tolerance between groups, both WT and Ulk1-iMKO mice had significantly lower body weight compared to respective sedentary groups following exercise training (Figure 1H).

To gain mechanistic insight into the role of Ulk1 in skeletal muscle in glucose clearance we subjected mice to insulin injection and measured Protein Kinase B/Akt phosphorylation at S473 (Shao et al., 2000; Cui et al., 2020). We harvested plantaris skeletal muscles prior to and 10 min after I.P. injection of 5 U/kg insulin. In WT mice, exercise training resulted in a significant increase in phosphorylation of the insulin receptor substrate Akt at S473 following insulin injection (Figures 1I,J), indicative of improved insulin response in skeletal muscle (Shao et al., 2000; Cui et al., 2020). However, there was no change in phosphorylation of Akt at S473 in plantaris muscles post-insulin injection in Ulk1-iMKO mice (Figures 1I,J). These data suggest that Ulk1 in skeletal muscle is required for exercise training-mediated improvement of insulin response.

Ulk2 is 55% homologous to Ulk1 and has been demonstrated to be redundant to Ulk1 in some aspects of autophagy/mitophagy regulation within some systems (Yan et al., 1999; Hara et al., 2008; Chan et al., 2009; Lee and Tournier, 2011), though recent evidence suggests more is different than similar between Ulk1 and Ulk2 (Demeter et al., 2020). We subjected *Ulk2*^–/–^ mice to voluntary running for 6 weeks, as we had done with Ulk1-iMKO mice. Ulk2^–/–^ mice has similar daily running distance and significantly improved endurance exercise capacity compared to sedentary mice, as evidenced by increased endurance capacity and reduced blood lactate (Figures 2A–C), similar to that of Ulk1-iMKO and their WT littermates (Figures 1B–D). Exercise training compared to sedentary counterparts resulted in marginal improvements in glucose clearance during GTT (Figures 2D,E), though body weight was significantly reduced post exercise training (Figure 2F).

To determine if the homologous Ulk2 is required for improved skeletal muscle insulin response post-exercise training, we again harvested plantaris skeletal muscle prior to and 10 min after I.P. injection of 5 U/kg insulin. In contrast to Ulk1-iMKO mice, insulin-stimulated S473-Akt phosphorylation was significantly increased in exercise trained Ulk2^–/–^ mice, indicative of improved skeletal muscle insulin response (Figures 2G,H), indicative of improved skeletal muscle insulin response. Taken together, these data suggest Ulk1, not Ulk2, is required for exercise training-induced improvement in skeletal muscle insulin response.

## Discussion

Regular exercise, particularly aerobic exercise, promotes overall health, in part, by improving mitochondrial quality within skeletal muscle. We have previously demonstrated that an essential mitophagy initiator, Ulk1, was required for improved glucose tolerance following exercise training (Laker et al., 2017). Herein, we have expanded upon our original findings and demonstrated that loss of skeletal muscle Ulk1 inhibits exercise training-induced improvements on insulin action in skeletal muscle, as evidenced by Akt S473 phosphorylation in response to a bolus intraperitoneal injection of insulin. Intriguingly, this important function of Ulk1 in skeletal muscle was not impaired in mice lacking Ulk2. Intriguingly, this important exercise training-induced metabolic adaptation in skeletal muscle was not impaired in Ulk2^–/–^ mice, even though these mice had marginal improvement in whole-body metabolic adaptation to exercise training. Ulk2 may function in tissues other than skeletal muscle in mediating the metabolic benefits of endurance exercise training.

Ulk1, the homolog of Atg1, initiates mitophagy following energetic stress when phosphorylated by AMPK at Ser555 (Egan et al., 2011; Laker et al., 2017; Saito et al., 2019). Activated Ulk1 promotes biogenesis of autophagosomes through initiating a cascade of events by complexing with and/or phosphorylating a number of downstream substrates (e.g., FIP200, Atg2, and Atg9) (Hosokawa et al., 2009; Mack et al., 2012; McAlpine et al., 2013; Papinski et al., 2014). Ulk proteins are known to exist in at least four variants, with Ulk1 and Ulk2 being the most similar with 55% homology, particularly in their C-terminal and N-terminal kinase regions (Hara et al., 2008; Chan et al., 2009). The degree of functional redundancy between Ulk1 and Ulk2 is not entirely clear as there are conflicting reports as to what degree Ulk2 acts like Ulk1 in regards to autophagy (Chan et al., 2007, 2009; Hara et al., 2008; Cheong et al., 2011; Demeter et al., 2020). In the present study, we showed that only loss of Ulk1, not Ulk2, impaired the exercise training-induced improvement in insulin-stimulated Akt activity in skeletal muscle. Ulk1 has been shown to be phosphorylated at Ser555 by Metformin *in vitro* (Egan et al., 2011), thus Ulk1 may play an important role in modulating beneficial metabolic adaptions.

It has been proposed that AMPK and Akt antagonistically regulate autophagy through Ulk1 (Bach et al., 2011). Ulk1 contains an Akt consensus motif and has been shown *in vitro* to be phosphorylated at Ser774 in response to insulin to inhibit autophagy (Bach et al., 2011). The loss of insulin-stimulated Akt phosphorylation in skeletal muscle of Ulk1-iMKO mice in the current study suggest that Ulk1 may have a role in reciprocally modulating Akt. This notion is supported by data describing a Ulk1-AMPK feedback loop where Ulk1 is able to modulate AMPK as evidenced by Ulk1-dependent phosphorylation sites on multiple AMPK isoforms (Loffler et al., 2011). Additionally, Ulk1 was recently shown to inhibit leucyl-tRNA synthetase 1 (LARS1), a key regulator of leucine metabolism and thereby protein synthesis, allowing available leucine to be used for ATP generation (Yoon et al., 2020). Taken as a whole in the context of our current findings, these results suggest a level of crosstalk between Ulk1-dependent autophagy and growth factor sensing mechanisms (e.g., Akt) that modulates the metabolic environment of skeletal muscle during exercise training. In sum, Ulk1 may be a viable target for interventions to treat metabolic dysfunction.

## Data Availability Statement

The raw data supporting the conclusions of this article will be made available by the authors, without undue reservation.

## Ethics Statement

The animal study was reviewed and approved by the Animal Care & Use Committee of University of Virginia.

## Author Contributions

JD designed the study, performed the experiments, analyzed the data, and wrote and edited the manuscript. RW and YG performed the experiments and edited the manuscript. MK designed the study and edited the manuscript. MZ performed the experiments and analyzed the data. ZY designed the study, analyzed the data, and wrote and edited the manuscript. All authors contributed to the article and approved the submitted version.

## Conflict of Interest

The authors declare that the research was conducted in the absence of any commercial or financial relationships that could be construed as a potential conflict of interest.

## Publisher’s Note

All claims expressed in this article are solely those of the authors and do not necessarily represent those of their affiliated organizations, or those of the publisher, the editors and the reviewers. Any product that may be evaluated in this article, or claim that may be made by its manufacturer, is not guaranteed or endorsed by the publisher.
